# Supplementary material for: Carbon removal support is tempered by concerns over whether biological methods are worth it
Source: Commun Earth Environ. 2025 Aug 28;6(1):711. doi: 10.1038/s43247-025-02654-x (PMC12394068; doi:10.1038/s43247-025-02654-x)
Supplement: Supplementary file 2 — Supplementary materials [file 43247_2025_2654_MOESM2_ESM.pdf]

# Supplementary materials

## Supplementary materials: contents


- Supplemental 1: Summary demographics
    - Table S1: Survey sample demographics, showing actual survey sample sizes and target quota sizes
    - Table S2: Workshop summary demographics

- Supplemental 2: Full survey protocol (for all the sections of the survey analysed and reported in this paper)

- Supplemental 3: Timeplan for the deliberative workshops (rooms 1 and 2)

- Supplemental 4: Full protocol for the deliberative workshops, containing full transcripts of all descriptions and introductions by facilitators, for the sections of the workshops analysed and reported in this paper

- Supplemental 5: workshop stimulus materials
    - 5a: Workshop slides, breakout room 1
    - 5b: Workshop slides, breakout room 2
    - 5c: Images of the CDR techniques discussed in the workshops
    - 5d: Copies of vignette cards

- Supplemental 6: Copy of questionnaires handed out during the workshop, for the 2nd iteration of the ranking task

- Supplemental 7: Additional graphs and data tables
    - Figure S1: Climate change worry
    - Table S3: Belief in anthropogenic climate change
    - Table S4: Net zero awareness
    - Table S5: Support for the goal of net zero emissions

## Supplemental 1: Summary demographics

*Table S1: survey demographics, showing actual survey sample sizes (n and %). Right-hand column shows target quota sizes, defined before distribution according to demographic statistics for the UK as a whole. Quotas not used for all characteristics for logistical reasons*

| | | **UK (N = 1978)** | | |
|---|---|---|---|---|
| | | **N** | **%** | **Quota (%)** |
| **Gender** | Male | 969 | 49.0 | 49.48 |
| | Female | 999 | 50.5 | 50.52 |
| | Non-binary / gender neutral | 5 | 0.3 | n/a |
| | Gender fluid | 1 | 0.1 | n/a |
| | Prefer not to say | 4 | 0.2 | n/a |
| | | | | |
| **Age** | 18 – 24 | 224 | 11.3 | 11.38 |
| | 25 – 34 | 393 | 19.9 | 19.32 |
| | 35 - 44 | 358 | 18.1 | 18.05 |
| | 45 – 54 | 377 | 19.1 | 19.41 |
| | 55 - 64 | 350 | 17.7 | 31.84 (55+) |
| | 65+ | 276 | 14.0 | |
| | | | | |
| **Education** | Post Graduate Qualification (e.g. MSc, PhD) / NVQ5 | 210 | 10.6 | n/a |
| | Bachelor's Degree / NVQ4 / Higher National Certificate / HND / Diploma in HE | 578 | 29.2 | n/a |
| | A Level / NVQ3 / SCE Higher Grade / Scottish cert. of sixth year studies / GNVQ Advanced Level | 488 | 24.7 | n/a |
| | NVQ 1 or NVQ 2 | 100 | 5.1 | n/a |
| | O-levels / General NVQ Foundation or Intermediate / GCSE / SCE Standard | 408 | 20.6 | n/a |
| | Apprenticeship; Other qualification (including vocational and work-related qualification) | 92 | 4.7 | n/a |
| | No academic or professional qualification | 73 | 3.7 | n/a |
| | Prefer not to say | 29 | 1.5 | n/a |
| | | | | |
| **Region** | Northern England (North West, North East, Yorkshire, Humber) | 458 | 23.2 | 23.17 |
| | Mid England (W Midlands, E Midlands, East of England) | 513 | 25.9 | 25.44 |
| | Southern England (South West, South East) | 451 | 22.8 | 22.20 |
| | Greater London | 232 | 11.7 | 13.48 |
| | Wales | 103 | 5.2 | 4.71 |
| | Scotland | 178 | 9.0 | 8.18 |
| | Northern Ireland | 43 | 2.2 | 2.81 |
| | | | | |
| **Ethnicity** | White British | 1728 | 87.4 | 86.0 |
| | Asian / Asian British | 120 | 6.1 | 7.50 |
| | Black / African / Caribbean British | 69 | 3.5 | 3.30 |
| | Mixed / Multiple ethnic groups | 25 | 1.3 | 2.20 |
| | Other | 21 | 1.1 | 1.0 |
| | Prefer not to say | 15 | 0.8 | n/a |
| | | | | |
| **Political affiliation** | Right-of-Centre (Conservative, Reform) | 400 | 20.2 | n/a |
| | Left-of-Centre (Labour, Lib Dem, Greens) | 953 | 48.2 | n/a |
| | Devolved parties (SNP, Plaid Cymru, DUP, Sinn Fein, Alliance) | 100 | 5.1 | n/a |
| | Undecided | 229 | 11.6 | n/a |

| | | | | |
|---|---|---|---|---|
| | Would not vote | 207 | 10.5 | n/a |
| | Prefer not to say | 73 | 3.7 | n/a |
| | Other | 16 | 0.8 | n/a |

*Table S2: workshop summary demographics*

| **Location** | | Cardiff | | Belfast | | London | | Edinburgh | |
|---|---|---|---|---|---|---|---|---|---|
| **Framing group** | | 1 | 2 | 1 | 2 | 1 | 2 | 1 | 2 |
| **Number** | *Total n = 60* | 7 | 8 | 6 | 7 | 8 | 7 | 8 | 9 |
| **Gender** | *Male* | 4 | 3 | 3 | 4 | 4 | 3 | 4 | 4 |
| | *Female* | 3 | 5 | 3 | 3 | 4 | 4 | 4 | 5 |
| **Age** | *18-24* | 1 | 1 | | | 1 | | 1 | |
| | *25-34* | 3 | 3 | 1 | 1 | | 1 | 3 | 3 |
| | *35-44* | | 2 | 2 | 2 | 2 | 1 | 1 | 2 |
| | *45-54* | 1 | 1 | 1 | 2 | 2 | 1 | 1 | 2 |
| | *55-65* | | 1 | | 1 | 1 | 2 | 2 | 1 |
| | *65+* | 2 | | 2 | 1 | 2 | 2 | | 1 |
| **Political affiliation** | *Centre-right* | | | | 1 | 1 | 1 | | |
| | *Centre-left* | 5 | 6 | 3 | 4 | 4 | 4 | 7 | 7 |
| | *Green* | 1 | 1 | 1 | | 2 | 1 | 1 | 1 |
| | *Other* | | | 2 | 2 | 1 | | | |
| | *Undecided* | | 1 | | | | | | 1 |
| | *Won't vote* | 1 | | | | | 1 | | |

# Supplemental 2: Survey protocol

**Information about the survey**

This survey is investigating opinions towards ways of tackling climate change. It should take around 15 minutes to complete. The survey is anonymous and your computer's IP address will not be recorded.

**What will I need to do?**
You'll be asked to answer some questions about your opinions on a variety of topics, and to respond to information we will provide within the survey. There are no right or wrong answers, so please answer as honestly as you can. At the bottom-right of the screen is an arrow button. Click on the arrow each time you are ready to move to the next page.

**Who is the survey for?**
The survey is being carried out by researchers at Oxford University and Manchester University. The survey is completely anonymous and your personal details will not be shared with anyone. Overall results may be used in reports, presentations and academic articles. If you have any questions or concerns about the survey, please contact Dr Emily Cox (emily.cox@smithschool.ox.ac.uk). The lawful basis for the processing of the data you provide is public interest.

**By clicking the button below, you acknowledge that your participation in the study is voluntary, you are 18 years of age or older, and you are aware that you may choose to terminate your participation in the study at any time.**

Consent Do you give your consent to take part in this study? Yes / No

Gender Please indicate your gender

Age What age bracket are you in?

Ethnicity Which of these options best describes your ethnicity?
- White British (1)
- Asian / Asian British (2)
- Black / African / Caribbean British (3)
- Mixed / Multiple Ethnic Groups (4)
- Other Ethnic Group (5)
- Prefer not to say (6)

Region What region of the UK do you live in?
- Northern England (North West, North East, Yorkshire and the Humber) (1)
- Mid England (West Midlands, East Midlands and East of England) (2)
- Southern England (South West and South East) (3)
- Greater London (4)
- Wales (5)
- Scotland (6)
- Northern Ireland (7)

Do you think humans are causing the climate to change?
- Definitely yes (1)
- Probably yes (2)
- Perhaps (3)
- Probably not (4)
- Definitely not (5)
- Don't know (6)

How worried are you about climate change? On a scale of 1 to 10, where 1 is not at all worried and 10 is the most worried.

Were you aware of the goal to reach 'net zero' emissions in the UK? Yes / No / Not sure

---

*Display This Question:*
*If Were you aware of the goal to reach 'net zero' emissions in the UK? = Yes*

Do you support the goal of net zero emissions?

- Strongly support (1)
- Slightly support (2)
- Neither support nor oppose (3)
- Slightly oppose (4)
- Strongly oppose (5)
- Not sure (6)

---

When you hear the term 'carbon removal', what are the first three words or images which come to mind?

- First word / image (1) ________________________________________________
- Second word / image (4) ________________________________________________
- Third word / image (5) ________________________________________________

You previously answered that ${Top-of-mind CDR/ChoiceTextEntryValue/1} is the first word or image which comes to mind when you hear the term 'carbon removal'. To you, is this word or image positive or negative?

- Very positive (1)
- Slightly positive (2)
- Neutral (3)
- Slightly negative (4)
- Very negative (5)

---

You previously answered that ${Top-of-mind CDR/ChoiceTextEntryValue/4} is the first word or image which comes to mind when you hear the term 'carbon removal'. To you, is this word or image positive or negative?

- Very positive (1)
- Slightly positive (2)
- Neutral (3)
- Slightly negative (4)
- Very negative (5)

---

You previously answered that ${Top-of-mind CDR/ChoiceTextEntryValue/5} is the first word or image which comes to mind when you hear the term 'carbon removal'. To you, is this word or image positive or negative?

- Very positive (1)
- Slightly positive (2)
- Neutral (3)
- Slightly negative (4)
- Very negative (5)

**Please carefully read the following explanation of carbon removal:**

Carbon removal is included in many strategies to meet 'net zero' emissions and tackle climate change. The idea is to remove excess carbon dioxide (CO2) from the atmosphere through various biological, chemical or physical processes. After being captured, the carbon dioxide would be stored by plants, in soils, or deep underground and in the deep ocean so that it cannot contribute to an increase in the Earth's temperature.

There are many ways of removing carbon. Some are quite widespread today - such as planting trees and restoring habitats.

Meanwhile others - such as capturing carbon directly from air - are only at a very early stage of development and only used at a very limited scale.

Before today, how much if anything would you say that you know about carbon removal?

- Know a great deal (1)
- Know a moderate amount (2)
- Know quite a lot (3)
- Know just a little (4)
- Know nothing (5)
- Never heard of it before (6)

How do you feel about carbon removal?

- Very positive (1)
- Slightly positive (2)
- Neither positive nor negative (3)
- Slightly negative (4)
- Very negative (5)

Please explain your choice regarding how you feel about carbon removal (open-ended)

When you think about the idea of carbon removal, to what extent to you feel the following emotions?

| | Very much (1) | Quite a lot (2) | Somewhat (3) | Not very much (4) | Not at all (5) |
|---|---|---|---|---|---|
| Worry (1) | ○ | ○ | ○ | ○ | ○ |
| Annoyance (3) | ○ | ○ | ○ | ○ | ○ |
| Aversion (4) | ○ | ○ | ○ | ○ | ○ |
| Satisfaction (5) | ○ | ○ | ○ | ○ | ○ |
| Powerlessness (6) | ○ | ○ | ○ | ○ | ○ |
| Hope (8) | ○ | ○ | ○ | ○ | ○ |
| Calmness (9) | ○ | ○ | ○ | ○ | ○ |
| Fear (10) | ○ | ○ | ○ | ○ | ○ |
| Neutral (2) | ○ | ○ | ○ | ○ | ○ |

How much would you say you know about the following different proposals for removing carbon from the atmosphere

| | Know a great deal (1) | Know quite a lot (2) | Know a moderate amount (3) | Know just a little (4) | Know nothing (5) | Never heard of before (6) |
|---|---|---|---|---|---|---|
| Capturing carbon directly from the air (DACCS) (1) | ○ | ○ | ○ | ○ | ○ | ○ |
| Bioenergy with Carbon Capture and Storage (BECCS) (2) | ○ | ○ | ○ | ○ | ○ | ○ |
| Planting trees (afforestation) (3) | ○ | ○ | ○ | ○ | ○ | ○ |
| Increasing carbon content of soil (Soil Carbon Sequestration) (4) | ○ | ○ | ○ | ○ | ○ | ○ |
| Restoring peatlands (10) | ○ | ○ | ○ | ○ | ○ | ○ |
| Wood in construction (5) | ○ | ○ | ○ | ○ | ○ | ○ |
| Speeding up the weathering of rocks (Enhanced Weathering) (6) | ○ | ○ | ○ | ○ | ○ | ○ |
| Burying biochar (7) | ○ | ○ | ○ | ○ | ○ | ○ |
| Increasing alkalinity of ocean water (ocean alkalinisation) (8) | ○ | ○ | ○ | ○ | ○ | ○ |
| Restoring coastal ecosystems ('blue carbon') (9) | ○ | ○ | ○ | ○ | ○ | ○ |

How do you feel about these proposals?

| | Very positive (1) | Fairly positive (2) | Neither positive nor negative (3) | Fairly negative (4) | Very negative (5) | Don't know enough about it (6) |
|---|---|---|---|---|---|---|
| Capturing carbon directly from the air (DACCS) (1) | ○ | ○ | ○ | ○ | ○ | ○ |
| Bioenergy with Carbon Capture and Storage (BECCS) (2) | ○ | ○ | ○ | ○ | ○ | ○ |
| Planting trees (afforestation) (3) | ○ | ○ | ○ | ○ | ○ | ○ |
| Increasing carbon content of soil (Soil Carbon Sequestration) (4) | ○ | ○ | ○ | ○ | ○ | ○ |
| Restoring peatlands (10) | ○ | ○ | ○ | ○ | ○ | ○ |
| Wood in construction (5) | ○ | ○ | ○ | ○ | ○ | ○ |
| Speeding up the weathering of rocks (Enhanced Weathering) (6) | ○ | ○ | ○ | ○ | ○ | ○ |
| Burying biochar (7) | ○ | ○ | ○ | ○ | ○ | ○ |
| Increasing alkalinity of ocean water (ocean alkalinisation) (8) | ○ | ○ | ○ | ○ | ○ | ○ |
| Restoring coastal ecosystems ('blue carbon') (9) | ○ | ○ | ○ | ○ | ○ | ○ |

## Supplemental 3: Deliberative timeplan

| Time | M | Task | Purpose |
|---|---|---|---|
| **10:00** | **15** | Coffee and consent forms | Greet participants, sign consent forms, allow for late arrivals |
| **10:15** | **5** | Introductions | Introduce the project team and aims<br>Check consent to record<br>Housekeeping |
| **10:20** | **5** | **Split into breakout groups** | |
| **10.25** | **20** | Icebreaker – drawing task | Participants asked to draw "something which represents their country / their local area"<br>Followed by discussion of their drawings |
| **10.45** | **30** | Presentation & net zero / carbon removal discussion | **Group 1:** Presentation on GGR<br>Ranking task – 4 scenarios for reductions vs removals<br>Discussion about the task and the presentation<br><br>**Group 2:** Presentation mixed in with discussion questions on net zero<br>Ranking task – 4 scenarios for reductions vs removals<br>Discussion about the task about CDR in relation to local issues / everyday life |
| **11:15** | **15** | **Break** | |
| **11.30** | **15** | AI exercise | Discuss and decide what question to ask the AI |
| **11:45** | **65** | Vignette task & discussion<br>Part 1<br>GGR techniques 1 & 2 | Techniques introduced in a random order<br>Hand round GGR objects and interact with them, ask participants for first associations<br><br>Read the card (2-3 mins)<br>Images on screen<br><br>Discussion:<br>• Identify salient issues for specific GGR technique<br>• Breakout 2: Emphasise participant-led issue identification |
| **12:50** | **30** | **Lunch** | |
| **1:20** | **30** | Vignette task & discussion<br>Part 2<br>GGR technique 3 | Techniques introduced in a random order<br>Hand round GGR objects and interact with them, ask participants for first associations<br><br>Read the card (2-3 mins)<br>Images on screen<br><br>Discussion:<br>• Identify salient issues for specific GGR technique<br>• Breakout 2: Emphasise participant-led issue identification |
| **1.50** | **15** | General Discussion: cross-cutting topics | General discussion seeking to looking across technologies |
| **2.05** | **25** | Ranking exercise | Lone working questionnaire<br><br>Group discussion: discuss and agree:<br>• Percentage CDR vs emissions reductions (4 scenarios) |

| | | | |
|---|---|---|---|
| | | | • How much each of the 4 GGR techniques should be used (using sticky dots)<br><br>Choose spokesperson(s) for final plenary |
| **2:30** | **15** | **Break** | |
| **2.45** | **35** | AI text<br>Lone working + discussion | Participants read the AI-generated text (5 mins)<br>Discussion of AI-generated text: does the text raise any issues that they weren't previously aware of? |
| **3:20** | **5** | **Back to plenary** | |
| **3.25** | **15** | Plenary discussion | All groups / participants discuss the day<br>Each breakout group presents their agreed recommendations for GGR implementation from the ranking exercise<br><br>Plenary discussion - What have they learnt?<br>How do they feel? |
| **3.40** | **10** | Q&A | Q&A for the facilitators<br>Placed at the end to avoid steering the earlier discussions |
| **3.50** | **5** | Honorarium & close | Thank participants for their time, informs them of next steps<br>Arrange payment |

# Supplemental 4: Full workshop protocol

### 10:15 – 10:20 [5 mins] Introductions [Plenary]

**(Begin recording AFTER recorders are introduced verbally)**

Hello everyone & welcome – thanks so much for coming today! We really appreciate you giving up your time to take part in this research, which we hope will be an enjoyable and interesting process for everyone.

### First Slide: who we are and aims

This project is being run by **[redacted]**. Our backgrounds are in social science. We'll just quickly introduce the project team [go round saying 1 sentence about ourselves].

We'll be co-ordinating this workshop and hopefully getting some really interesting discussions going. If at any point you need anything, just grab one of us – we have yellow dots on our badges so you can identify us. We are independent researchers, we're not here to sell anything or to try and convince you of anything, we're just interested in your honest views. We are funded by the Natural Environment Research Council, which is a publicly-funded organisation supporting scientific research in the UK.

So, we've invited you here today to participate in a workshop, to explore what you think about science and society. We kept the topic deliberately vague before today, because we didn't want you to do any preparations, but now that you're here we can tell you that we're particularly interested in climate change and in proposals for meeting the goal of net zero emissions. We are going to be discussing ideas for how to get to net zero, and particularly for something called carbon removal, which is basically removing carbon dioxide which has already been emitted back out from the atmosphere. Don't worry if you don't feel like you know anything about this stuff, or if what I just said made no sense to you; we still want your opinions, and we'll be providing information as we go along.

Most importantly, there are **no right or wrong answers** - we are interested in how YOU see things – what you think and feel, what you want/don't want and like/don't like. So you don't have to have any special knowledge – just be yourself.

We are of course happy to try to answer any of your questions, and we really want to know what questions you have. **We may ask you *why*** you are asking a question or ask you to expand on it; this doesn't mean we think it's a silly question, or that we're trying to avoid answering, but are genuinely interested in your thoughts.

One word about **confidentiality**—what you say in this room will be kept anonymous — you will not be identified or named in any of our resulting research. The recorders are just for making sure that it gets typed up correctly. After it's all been transcribed, your names will be changed to 'aliases' or '**pseudonyms'** so you can't be identified.

We also need you to keep what is said here confidential. You can talk about today with friends and family- but please don't identify people who said particular things. On a similar note, please don't record any of today's session on your mobile phones or things like that.

Could I ask that all phones are put on silent or turned off. Thank you.

Also, do speak up, and please try not to speak over one another, as this makes it very difficult to decipher what people are saying when we listen to the tapes. Can I just check that everyone is happy and comfortable with all this?

OK so the first thing we'd like to do is show you all a short video. After that, we'll split you into breakout groups. This is because it's difficult with such a big group to get a good discussion going where everyone gets chance to speak.

This video was made by our colleagues at **[redacted]**. It explains a bit about our project and the things we're working on. We're not going to discuss all the topics raised in the video today; it's just to give you some context. This video uses the term 'GGR', which stands for 'greenhouse gas removal'. But I don't really like using acronyms, so we're just going to call it carbon removal. They're basically the same thing.

**Slide: video**

Now for breakout groups, where we can start the tasks and the discussion. You have already been randomly asigned to a breakout group, according to the sticky dots on your name badges. A member of our team will be in each group to facilitate the discussions.

If you have a [colour] dot, you're in Group 1 and you'll be staying in here. If you have a [colour] dot, you're in Group 2 – please follow [name] to the [room].

***Session of general discussion of climate change and CDR – see slides***

**Next slide: four scenarios.**

Before we start the discussion, I'd like to ask you to think about this question up on the slide - how much in total do you think we in [the UK / Scotland / Wales / Northern Ireland / your local area] should reduce emissions, versus how much we should remove carbon from the atmosphere?

You could think of all the possible options there are for reducing emissions: things like reducing how much energy we use, switching to renewables, changing our diets, changing our industry etc. Alternatively, we could do lots and lots of carbon removal, which means we would require fewer changes to our lifestyles and our economies to cut emissions.

***Slide animations: click through***. So here on the slide are four possible scenarios:

We could:

- Focus on reducing emissions, and not use carbon removal at all
- Reduce emissions by 90%, and use carbon removal for 10% of emissions
- Reduce emissions by 75%, and use carbon removal for 25% of emissions
- Reduce emissions by 50%, and use carbon removal for 50% of emissions

We would like you to discuss between yourselves, and decide as a group which scenario you would prefer.

Now, obviously we haven't given you very much information about these ideas so far! Don't worry, we're going to give you much more detail and more time for discussion later in the workshop, and

we'll come back to this question again later as well. For now, we're just interested in your 'gut feeling' about this question.

**4 mins – group discussion**

Could I get a show of hands – which scenario would you choose? Who would choose scenario 1? Scenario 2? Etc

**Facilitator notes show of hands**

### 11:45 – 12:50 [65 mins]: Vignette Task

For the next session, we're going to look in a little more detail at some specific proposals for carbon removal techniques. We're going to focus on three techniques for carbon removal. We'll do two techniques before lunch, and one after lunch.

*Technique chosen randomly, to minimise ordering effect*

First, we have some objects for you to pass round, which represent each technique.

- Peat- jar is passed around the table. Each participant is asked to sniff the peat and give their first association, it can be anything!
- Biochar sample is passed around the table. Each participant is asked to roll the char around in their hand and give their first association, it can be anything!
- Perennial bioenergy crop samples are passed around. Each participant is asked to give their first association, it can be anything!

**[Vary direction and starting point in the group]**

Now, we're going to give you a card with some information about this technique. I'd like you to take a few minutes to read the card.

**Observers pass round vignette cards**

In order to provide you with some visual ideas of what these techniques look like, we're also going to put some images up on the screen. Now, there are many possible images for what these techniques might look like in practice, and if we choose the images ourselves then it might leave it open to bias from us, so instead we've used a google image search to look for images. We carried out this google search in advance, to make sure that both breakout groups are seeing the same images.

**Slide: google image results for technique #1**

*Google images suggested search terms:*

- *"Peatland restoration -diagram"*
- *"Biochar -diagram"*
- *"Perennial bioenergy crops -diagram"*

**2-3 minutes quiet reading time**

### 11:50 – 12:20: Vignette #1

- Room 1: general discussion with prompt questions e.g.:

  - [High priority] Thinking about the first association you had when [e.g. sniffing the peat], have your associations changed after finding out more about this [peatland restoration/biochar/bioenergy crops]? How did you feel at first and how do you feel now about [peatland restoration/biochar/bioenergy crops] now?
  - [High priority] Are there things which you might find **controversial** about this technique? **Why?**
    - How about your friends, neighbours etc – might they find something controversial about this technique?
  - Are there issues which are specific to [Scotland / Wales / N. Ireland / UK]?
  - Would this technique work on a global level?
    - Would this help the world to meet its climate targets?

- Room 2: participant-led discussion
  - [High priority] Thinking about the first association you had when [e.g. sniffing the peat], have your associations changed after finding out more about this [peatland restoration/biochar/bioenergy crops]? How did you feel at first and how do you feel now about [peatland restoration/biochar/bioenergy crops] now?
  - [High priority] Are there things which you find potentially **controversial** about this technique? **Why?**
    - How about your friends, neighbours etc – might they find something controversial about this technique?
  - [priority] Would this technique work in [local area]? **Why / why not?**
  - Can you imagine living near to a site where [peatland restoration/biochar/bioenergy crops] was being implemented to remove carbon from the atmosphere? **What would that be like?**
  - Besides removing carbon dioxide, can you think of any other effects of implementing **[peatland restoration/biochar/bioenergy crops]**?

**For each response, facilitator asks 'what makes you say that?' or 'what makes you interested to ask that?', and tries to bring in other members of the group as well**

### 12:20-12.50: Vignette #2

Now I'd like us to do the same thing again, but looking at a different technique this time.

Once again, we'll pass round some objects for you to pass around first **[see above for instructions on how to interact with the objects]**

**Observers pass round vignette cards**

As before, we'll give you a card with information on the technique; please spend 2-3 minutes reading the information on the card. Whilst you're doing this, we will also put some images on the screen.

**2-3 minutes quiet reading time, followed by discussion – see above for prompt questions**

### 1:20 – 1:50 [30 mins]: Vignette task continued

**Observers Re-start recorders**

Before we start the next session, could we just go around the room again and say our names nice and clearly. This is because it helps our transcribers to know who is saying what when they type it up. I'll start...

*Go around the group, starting with facilitators, just saying names this time. Facilitators add 'facilitating'*

This session will be the same as the previous ones, but with one more carbon removal technique for us to discuss.

Once again, we'll be handing out objects and cards. We'll pass round the object and get your thoughts on it, and then we'll have a couple of minutes to read the card and look at the images on the screen, and about 20 minutes discussing.

So first let's pass round the object...

**Observers pass round vignette cards**

2 mins reading time

*See above for prompt questions*

## 1:50 – 2:05 [15 mins] General discussion: cross-cutting topics

### Next slide (holding slide), to avoid having images of one technique up on the screen during this session

Now to wrap up that session, I'd like you to think about all the three techniques we've just been talking about. I'll leave the cards and the objects on the table to jog your memories. I'd like us to discuss all three techniques.

**Room 1 prompt questions:**

- [High priority] Thinking about the discussions on peatland restoration, biochar and perennial bioenergy crops, did you notice any **controversies** which were similar across the techniques?
    - Can you elaborate on that?
    - Did anyone else notice this topic/concern/matter?
    - Do you trust that experts are able to address this topic/concern/matter?
    - Do you think communities have a role to play in this topic/concern/matter?
    - Were any one of the three techniques more or less controversial than the others? **Why?**
- Did any of the three proposals feel more or less realistic to you? **Why?**
    - What is it about technique X that seems less realistic?
- Are there issues which relate specifically to [Scotland / Wales / N. Ireland / UK]?
    - What makes you mention that? How do you feel about it?
- Did any of them appear more or less risky? In what ways/**why?**

**Room 2 prompt questions:**

- [High priority] Thinking about the discussions on peatland restoration, biochar and perennial bioenergy crops, did you notice any **controversies** which were similar across the techniques?
    - Can you elaborate on that?
    - Did anyone else notice this topic/concern/matter?
    - Do you trust that experts are able to address this topic/concern/matter?
    - Do you think communities have a role to play in this topic/concern/matter?
    - Were any one of the three techniques more or less controversial than the others? **Why?**

- Did any of the three proposals feel more or less realistic to you? **Why?**
    - What is it about technique X that seems less realistic?
- Are there issues which relate specifically to your local area?
    - What makes you mention that? How do you feel about it?
- Did any of them appear more or less risky? In what ways/**why?**

### 2:05 – 2:30 [25 mins] Ranking exercise

**Stay on holding slide for now**

Now we're going to do a task which involves deciding how much of these techniques we should use overall.

We're handing round a very short questionnaire for you to complete individually. **Observers hand round questionnaire**

First on the questionnaire is the same question we did right at the start, about how much we should reduce emissions versus removing them using carbon removal techniques. Just like before, I would like you to think of all the possible options there are for reducing emissions – things like reducing how much energy we use, switching to renewables, changing our diets, changing our industry and so forth. Alternatively, we could do lots and lots of carbon removal, which means we would require fewer changes to our lifestyles and our economies to cut emissions, but would have to deal with all the challenges to carbon removal which we talked about previously.

So the first question on the questionnaire is, how much in total should we remove carbon from the atmosphere, instead of reducing it? When we say 'we', we're mainly referring to [Scotland / Wales / Northern Ireland / UK / the local area].

We've given you the same four scenarios to choose from; please pick one. It doesn't have to be the same scenario as you chose earlier – you can change your mind, or stay the same, it's up to you.

Please read the questionnaire and take a couple of minutes to respond by yourselves to both of the questions on there. Don't forget to write your names at the top, and also please write that you're in [Group 1 / 2]

**2 minutes – filling out ranking questionnaire**

**Slide: Four scenarios**

Now we're going to discuss those same questions as a group.

The first question was, how much in total should we remove carbon from the atmosphere, instead of reducing it? Here are the four scenarios on the slide again.

We would like you to discuss between yourselves, and decide as a group which scenario you would prefer.

*If needed, prompt by asking people to volunteer what they wrote on their questionnaire. Do others agree?*

**4 mins – group discussion**

Have we reached an agreement? What would you choose?

**Why?**

**2:20 latest: move on to next task**

**Slide: How much of a role should each of the four techniques play?**

OK great, thank you. Next, I would like you to decide how much of a role each of the three techniques we discussed earlier should play. **Observers to hand out sticky dots**

On the table are the same cards we used earlier, with each of the three carbon removal techniques printed on. There's no need to re-read the card, as it's just the same info we had before. As a group, we're going to give you 9 sticky dots. Please decide as a group where to place your sticky dots.

So for example, if you like the three techniques equally, you would place three sticky dots on each. Whereas if you think only one should be used, and the others not at all, you could place all 9 on that one technique, and leave the others blank. Or, any other combination you wish!

Does that make sense? **[Approx. 10 minutes for exercise**]

**Observers make sure there's a recorder in the middle of the group for the discussion**

Now finally, we have one more thing for you to decide before we go to the break. Later on this afternoon, we'll go back into the big group, and I'd like us to feed back our results from this exercise to the others. So can you please choose one person to act as a spokesperson for the rest of the group? Their job will be to say to the other group what the final results of your ranking exercise were – which of the carbon removal percentages you chose, and how much you felt each of the three techniques should be used.

**Facilitators ensure that a spokesperson has been chosen before going to the break**

**Observers collect completed questionnaires**

# Supplemental 5: Workshop stimulus materials

## 5a: workshop slides, room 1 'techno-economic' framing

### Climate scenarios

- You might be aware that the earth's climate is changing
- Experts predict that global average temperatures could increase by 2.7 degrees Celsius, if we don't rapidly address climate change
  - Impacts include heatwaves, drought, storms, and sea level rise
  - Across the UK, climate change will mean more chance of heatwaves and flooding

### Reaching 'net zero' emissions

- The UK government has set a target of 'net zero' emissions by 2050, in order to end the UK's contribution to climate change.
- The UK and Northern Ireland each have targets for net zero by 2050. Scotland's target is for net zero by 2045. Wales has a target for 95% reduction in emissions.
- The different targets reflect differences in the regions and the sectors which make up their economies

### Reducing the carbon emissions we produce

There are many things we can do to reduce emissions and get towards net zero

These could include shifting to renewable forms of energy, reducing how much energy and goods we consume, changing our diets, and changing our transport habits.

### Removing carbon from the atmosphere

- Many scientists now agree that **in addition** to all this, we will have to actually remove some carbon dioxide from the atmosphere

This is mainly to compensate for things which are difficult to decarbonise, like farming, shipping, aviation and industry

Net zero means that whatever emissions are being produced, they are balanced by actions to remove greenhouse gases from the atmosphere

### Fundamental questions over carbon removal

1. Experts do not agree on how much carbon removal we should do
2. Big uncertainties about the cost of different techniques
3. Uncertainties about effectiveness of techniques, and how long they would take to work
4. Debate over which kinds of organisations should be responsible, and who should be accountable
5. Other locally-important issues, depending on how and where it is done

CORE

### How much in total should we remove carbon from the atmosphere, instead of reducing it?

We could:

- Focus on reducing emissions, and not use carbon removal at all
- Reduce emissions by 90%, and use carbon removal for 10% of current emissions
- Reduce emissions by 75%, and use carbon removal for 25% of current emissions
- Reduce emissions by 50%, and use carbon removal for 50% of current emissions

**We're going to discuss this topic in more detail throughout the workshop; for now, we're just interested in your 'gut feeling'**

CORE

## 5b: workshop slides, room 2 'everyday life' framing

Unlike in room 1, these slides were structured as a discussion with participants, with a back-and-forth style and prompt questions to encourage participants to frame the topic in their own way and to connect it to their everyday life

### 'Net zero' futures

- We are interested in climate change because of the impacts it might have on our future way of life
- Are you familiar with the concept of 'net zero'?
- What might everyday life in a 'net zero' world look like?

### Reducing the carbon emissions we produce

There are many things we can do to reduce carbon emissions and get towards net zero.

These could include shifting to renewable forms of energy, reducing how much energy and goods we consume, changing our diets, and changing our transport habits.

Can anyone give an example of a change like this in their community? What sort of impact did it have?

### Removing carbon dioxide from the atmosphere

- Many scientists now agree that **in addition** to all this, we will have to remove and lock away some carbon dioxide from the atmosphere.
- This is because some important activities may be hard to change: such as farming, shipping and aviation.

### Questions about carbon removal for 'net zero'

1. Experts do not agree on how much carbon removal we should do
2. Big uncertainties about the cost of different techniques
3. Uncertainties about effectiveness of techniques, and how long they would take to work
4. Debate over which kinds of organisations should be responsible, and who should be accountable
5. Other locally-important issues, depending on how and where it is done

### How much in total should we *remove* carbon from the atmosphere, instead of *reducing* it?

We could:

- Focus on reducing emissions, and not use carbon removal at all
- Reduce emissions by 90%, and use carbon removal for 10% of emissions
- Reduce emissions by 75%, and use carbon removal for 25% of emissions
- Reduce emissions by 50%, and use carbon removal for 50% of emissions

**We're going to discuss this topic in more detail throughout the workshop; for now, we're just interested in your 'gut feeling'**

## 5c: technique images (pre-defined images search)

When providing visual imagery of the three CDR techniques, we used a Google Image search to avoid inadvertently exerting too much researcher influence over the images used. Images are crucial for improving engagement and providing an informational basis for non-experts to discuss complex or novel topics. However, researcher framings – particularly images – can also exert a substantial impact on participant discourses in deliberative research settings. Previous research has often reflected on this, but has generally stopped short of proposing concrete measures for countering it in research design. In addition, the Google Image approach enabled us to show a broad range of images on the screen, rather than just one.

We ran the Google search in advance, to ensure that all groups saw the same images, using the search terms [technique] -diagram (see below). During piloting, we noticed that search terms were resulting in a large number of complex and technical diagrams of the techniques, rather than any indication of what they might look like in the real world. Including -diagram removed these technical images.

Unfortunately, for copyright reasons, we are unable to include the images which were provided by the Google Image search. We did not filter the images by license – this was attempted during the research design, but did not deliver enough relevant results. Therefore the images have been redacted for publication, although search terms and website titles of hits are shown.

### Peatland restoration:

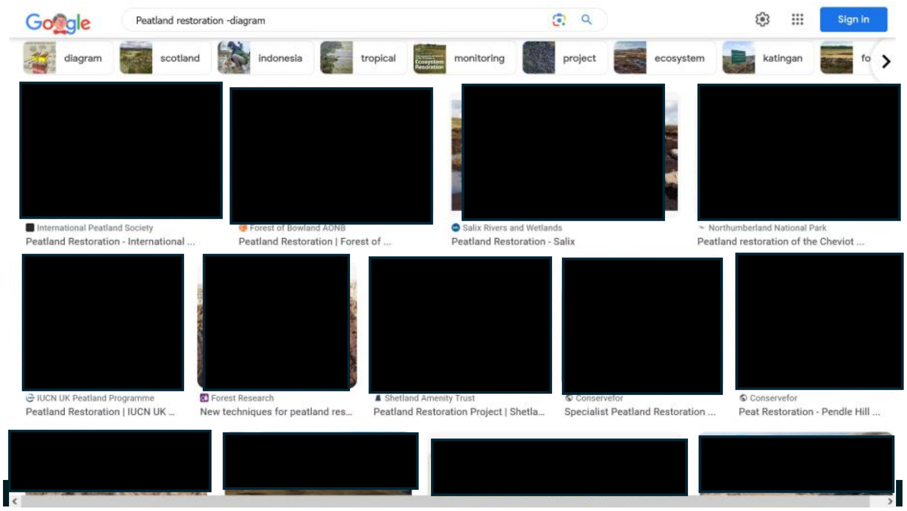

## Biochar:

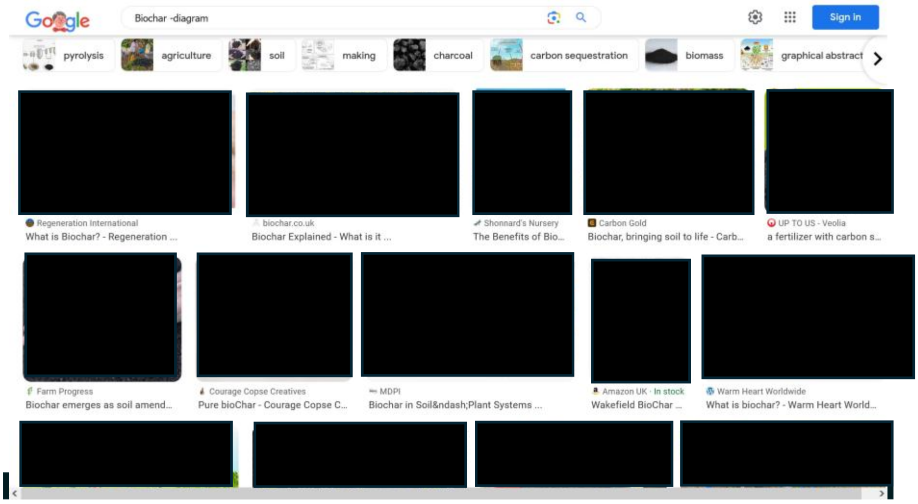


## Perennial biomass crops:

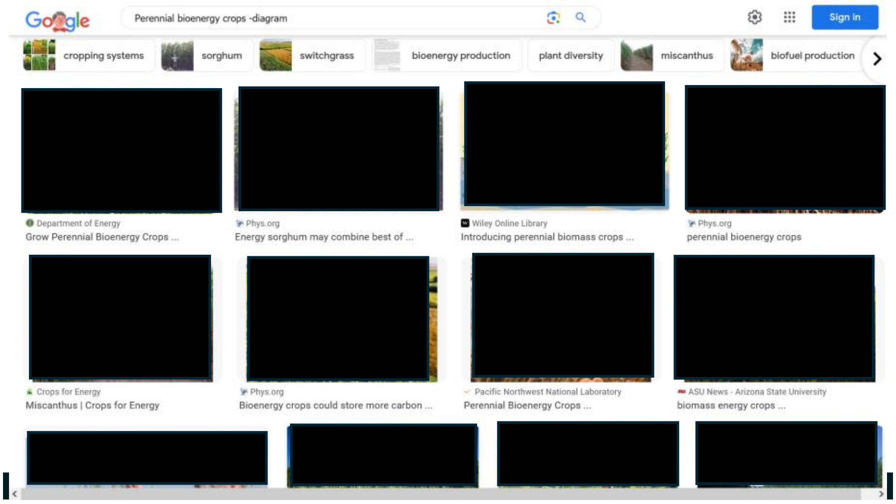

## 5d: vignette cards

Room 1:

# Perennial biomass crops

Perennial biomass crops are fast-growing plants, such as miscanthus or willow, which absorb carbon as they grow. The plants store carbon in their roots, and the roots stay in the soil between each harvest. The plants themselves can then be burnt to generate electricity and/or heat. The carbon released when the plants are burned would be captured and stored deep underground.

Cultivated across 10% of total UK cropland, this would store 2.8Mt $CO_2$ per year, less than 1% of current UK emissions. If the plants were burnt and the $CO_2$ stored underground, this technique could remove up to 53Mt $CO_2$ per year, around 15% of current UK emissions, while also providing renewable energy.

Some experts are concerned that perennial biomass crops would need a lot of land. Clearing forest or other carbon-rich habitats to make way for bioenergy crops, or importing crops from abroad, would lead to further emissions.

Room 2:

# Perennial biomass crops

Perennial biomass crops are fast-growing plants, such as miscanthus or willow, which absorb carbon as they grow. The plants store carbon in their roots, and the roots stay in the soil between each harvest. The plants themselves can then be burnt to generate electricity and/or heat. The carbon released when the plants are burned would be captured and stored deep underground.

Perennial crops could be grown on land not currently used for food crops. They can prevent erosion, and might provide new sources of revenue for farmers and communities. If burnt and the $CO_2$ is then stored underground, the crops can generate renewable, carbon-negative energy.

Some communities are concerned that biomass crops need a lot of land, which could otherwise be used for housing or conservation. Farmers would have to change the types of crops they grow and change their land management practices.

Room 1:

# Peatland Restoration

Peatland restoration involves rewetting peat soils which have been drained and degraded. Drained peat soils are highly fertile, but emit lots of $CO_2$; the goal of restoration is to reduce emissions, and ideally enable peatlands to absorb more CO2 in the future.. Peat soils cover 12% of the UK, but around 80% of these are degraded today.

Peat restoration could help to reduce UK emissions by 8Mt per year – that's about 2% of current emissions. The carbon is stored directly in the peat soil, which means if the land is disturbed or degraded in the future, this carbon would quickly return to the atmosphere.

Some experts are concerned that peat restoration is a slow, difficult process, and that techniques for measuring and certifying actual carbon removal are very uncertain.

Room 2:

# Peatland Restoration

Peatland restoration involves rewetting peat soils which have been drained and degraded. Drained peat soils are highly fertile, but emit lots of $CO_2$; the goal of restoration is to reduce emissions, and ideally enable peatlands to absorb more CO2 in the future.

Peat restoration could restore previously degraded land, and could help local economies, for instance by helping farmers to grow alternative crops whilst also earning extra money by selling carbon credits.

Some communities are concerned that restoration would be incompatible with other well-established ways of making a living from peat, whether linked to conventional farming, leisure or other activities.

Room 1:

# Biochar

Biochar is a type of charcoal which is created when plant material is heated at high temperature without oxygen using a technique called pyrolysis, using a pyrolysis facility or machine. The biochar retains the $CO_2$ which the plants have absorbed, and is added to the soil, storing the carbon.

The carbon storage potential of biochar is still uncertain, but one estimate is up to 15Mt $CO_2$ per year in 2050, which is 4% of total UK emissions. The storage could potentially be long-term and stable, because the biochar remains bound in the soil for decades or centuries. Biochar may also help crops by providing nutrients to plants.

Some experts are concerned that plant responses to biochar are poorly understood – in some studies, the crop yields have actually decreased. There is a trade-off between the carbon removal potential and the benefit to the plants, and the carbon removed can be difficult to measure and verify.

Room 2:

# Biochar

Biochar is a type of charcoal which is created when plant material is heated at high temperature without oxygen using a technique called pyrolysis, with a pyrolysis facility or machine. The biochar contains the $CO_2$ which the plants have absorbed, and is added to the soil, storing the carbon.

Biochar could help crops to grow by providing nutrients to plants, reducing the acidity of soils, and improving the water content and drainage of the soil. It could help local economies, for instance by enabling farmers to grow food whilst also earning extra money by selling carbon credits.

Some communities are concerned that biochar will result in crops being grown just to be burnt for biochar, which could divert land away from other uses such as food or housing. There is uncertainty about the benefits biochar brings to crops.

## Supplemental 6: Ranking task individual questionnaire

**Your Name:**

**Your Group:**

1. How much in total should we remove carbon from the atmosphere, instead of reducing it?

Please tick **one** box

☐ Focus on reducing emissions, and not use carbon removal at all

☐ Reduce emissions by 90% and use carbon removal for 10% of current emissions

☐ Reduce emissions by 75% and use carbon removal for 25% of current emissions

☐ Reduce emissions by 50%, and use carbon removal for 50% of current emissions

2. Earlier, we discussed three techniques for carbon removal. How much of a role should each of them play in addressing climate change?

Please answer for each of the three techniques. Please choose just one response for each technique (three ticks in total)

| | Plays a major role | Plays somewhat of a role | Plays only a small role | Should not be used |
|---|---|---|---|---|
| Biochar – A type of charcoal created by burning organic material, buried in the soil | | | | |
| Perennial biomass crops – Growing large crops with deep roots, which store carbon in the soil and can be burnt for energy | | | | |
| Peatland restoration - Restoring peatlands so they absorb $CO_2$ rather than emitting it | | | | |

## Supplemental 7: Additional graphs and data tables

*Figure S1: Responses to the question "How worried are you about climate change? On a scale of 1 to 10, where 1 is not at all worried and 10 is the most worried."*

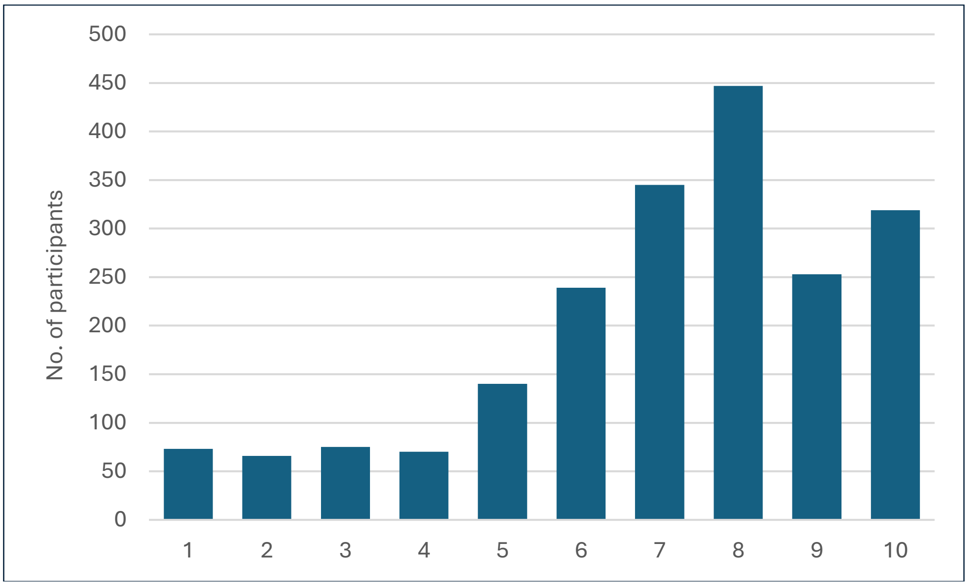


*Table S3: Responses to the question "Do you think humans are causing the climate to change?"*

| | | Frequency | Percent |
|---|---|---|---|
| Valid | Definitely yes | 1055 | 52.0 |
| | Probably yes | 601 | 29.6 |
| | Perhaps | 219 | 10.8 |
| | Probably not | 82 | 4.0 |
| | Definitely not | 60 | 3.0 |
| | Total | 2017 | 99.5 |
| Missing | | 10 | .5 |
| Total | | 2027 | 100.0 |

*Table S4: Responses to the question "Were you aware of the goal to reach 'net zero' emissions in the UK?"*

| | | Frequency | Percent |
|---|---|---|---|
| Valid | Yes | 1577 | 77.8 |
| | No | 237 | 11.7 |
| | Not sure | 213 | 10.5 |
| | Total | 2027 | 100.0 |

*Table S5: Responses to the question "Do you support the goal of net zero emissions?"*
*Only answered by those who previously said that they were 'aware' of the goal to reach net zero emissions in the UK (n=1577)*

| | | Frequency | Percent | Valid Percent |
|---|---|---|---|---|
| | Strongly support | 785 | 38.7 | 49.8 |
| | Slightly support | 456 | 22.5 | 28.9 |
| | Neither support nor oppose | 161 | 7.9 | 10.2 |
| | Slightly oppose | 75 | 3.7 | 4.8 |
| | Strongly oppose | 93 | 4.6 | 5.9 |
| | Not sure | 7 | .3 | .4 |
| | Total | 1577 | 77.8 | 100.0 |
| Missing | | 450 | 22.2 | |
| Total | | 2027 | 100.0 | |
