# Supplementary material for: Carbon removal support is tempered by concerns over whether biological methods are worth it
Source: Commun Earth Environ. 2025 Aug 28;6(1):711. doi: 10.1038/s43247-025-02654-x (PMC12394068; doi:10.1038/s43247-025-02654-x)
Supplement: Supplementary file 3 — Reporting summary [file 43247_2025_2654_MOESM3_ESM.pdf]

nature portfolio

Corresponding author(s): Emily Cox

Last updated by author(s): Jun 9, 2025

# Reporting Summary

Nature Portfolio wishes to improve the reproducibility of the work that we publish. This form provides structure for consistency and transparency in reporting. For further information on Nature Portfolio policies, see our Editorial Policies and the Editorial Policy Checklist.

## Statistics

For all statistical analyses, confirm that the following items are present in the figure legend, table legend, main text, or Methods section.

| n/a | Confirmed | |
|---|---|---|
| ☐ | ☒ | The exact sample size (*n*) for each experimental group/condition, given as a discrete number and unit of measurement |
| ☐ | ☒ | A statement on whether measurements were taken from distinct samples or whether the same sample was measured repeatedly |
| ☐ | ☒ | The statistical test(s) used AND whether they are one- or two-sided<br>*Only common tests should be described solely by name; describe more complex techniques in the Methods section.* |
| ☒ | ☐ | A description of all covariates tested |
| ☐ | ☒ | A description of any assumptions or corrections, such as tests of normality and adjustment for multiple comparisons |
| ☐ | ☒ | A full description of the statistical parameters including central tendency (e.g. means) or other basic estimates (e.g. regression coefficient) AND variation (e.g. standard deviation) or associated estimates of uncertainty (e.g. confidence intervals) |
| ☒ | ☐ | For null hypothesis testing, the test statistic (e.g. *F*, *t*, *r*) with confidence intervals, effect sizes, degrees of freedom and *P* value noted<br>*Give P values as exact values whenever suitable.* |
| ☒ | ☐ | For Bayesian analysis, information on the choice of priors and Markov chain Monte Carlo settings |
| ☒ | ☐ | For hierarchical and complex designs, identification of the appropriate level for tests and full reporting of outcomes |
| ☐ | ☒ | Estimates of effect sizes (e.g. Cohen's *d*, Pearson's *r*), indicating how they were calculated |

*Our web collection on statistics for biologists contains articles on many of the points above.*

## Software and code

Policy information about availability of computer code

| | |
|---|---|
| Data collection | No novel software or code was developed in this research |
| Data analysis | No novel software or code was developed in this research. Data was analysed using IBM SPSS statistics (version 25), and NVivo qualitative analysis software (version 14) |

For manuscripts utilizing custom algorithms or software that are central to the research but not yet described in published literature, software must be made available to editors and reviewers. We strongly encourage code deposition in a community repository (e.g. GitHub). See the Nature Portfolio guidelines for submitting code & software for further information.

## Data

Policy information about availability of data

All manuscripts must include a data availability statement. This statement should provide the following information, where applicable:

- Accession codes, unique identifiers, or web links for publicly available datasets
- A description of any restrictions on data availability
- For clinical datasets or third party data, please ensure that the statement adheres to our policy

All source data for this study is publicly available via the UK Data Service at https://reshare.ukdataservice.ac.uk/857507/ Dataset DOI: 10.5255/UKDA-SN-857507

## Research involving human participants, their data, or biological material

Policy information about studies with human participants or human data. See also policy information about sex, gender (identity/presentation), and sexual orientation and race, ethnicity and racism.

| | |
|---|---|
| Reporting on sex and gender | We do not conduct any analysis on sex or gender. Demographic data on gender was collected in the survey and focus groups, as reported in the demographic data tables in the Supplementary Materials. Categories used were male, female, non-binary, gender-fluid, prefer not to say. |
| Reporting on race, ethnicity, or other socially relevant groupings | We have not conducted any analysis on race, ethnicity or other socially relevant groupings. Demographic data on ethnicity was collected in the survey, as reported in the demographic data tables in the Supplementary Materials (Supplementary 1, Table S1), but these categories were not used as the basis for any analysis. |
| Population characteristics | See above |
| Recruitment | Participants were recruited using third-party recruitment companies, who use a mix of databases, contact lists and local recruiters. For the survey, recruitment was carried out by Qualtrics, using quotas to obtain a nationally-representative UK sample according to age, gender, ethnicity, and region of the UK. This was tested against census data to ensure the end sample reflected UK-wide demographics for these variables.<br><br>For the workshops, we hired a third-party recruitment company to recruit 18 participants per workshop from the general population in the four locations. We used quota sampling to ensure a roughly even mix of gender, age and ethnicity. We also attempted to recruit an even mix of political affiliation, by asking who they would vote for if a general election were held tomorrow, and who they voted for in the previous general election in 2019. However, it proved extremely challenging to recruit people with right-of-centre political affiliations, and all groups were underrepresented in this respect.<br><br>All these details are included in the Methods sections of the manuscript. |
| Ethics oversight | Ethical approval for this study was granted by Oxford University School of Geography and Environment research ethics committee, ethics approval no. SOGE C1A 23 78. |

Note that full information on the approval of the study protocol must also be provided in the manuscript.

# Field-specific reporting

Please select the one below that is the best fit for your research. If you are not sure, read the appropriate sections before making your selection.

☐ Life sciences ☒ Behavioural & social sciences ☐ Ecological, evolutionary & environmental sciences

For a reference copy of the document with all sections, see nature.com/documents/nr-reporting-summary-flat.pdf

# Behavioural & social sciences study design

All studies must disclose on these points even when the disclosure is negative.

| | |
|---|---|
| Study description | Mixed-methods study with 4 deliberative workshops in England, Wales, Scotland & Northern Ireland (8 breakout groups in total), and a UK-wide survey |
| Research sample | Workshops: 60 participants in total, using quotas (see below). Roughly 7-8 in each breakout group, after drop-outs (see below).<br>Survey = 2027 randomly recruited adults in the UK (see below for demographic quotas)<br>All particpants had to be over 18, with a good standard of spoken and written English.<br>For the workshops, participants had to have lived in the relevant area for 5 years minimum, to ensure that they could speak to the place-based discourses we wished to elicit. |
| Sampling strategy | Random sampling by a third-party recruitment company, using quotas to obtain a nationally-representative UK sample according to age, gender, ethnicity, and region of the UK, tested against census data to ensure the end sample reflected UK-wide demographics for these variables.<br>Workshops: quota sampling to ensure a roughly even mix of gender, age and ethnicity. We also attempted to recruit an even mix of political affiliation, by asking who they would vote for if a general election were held tomorrow, and who they voted for in the previous general election in 2019. However, it proved extremely challenging to recruit people with right-of-centre political affiliations, and all groups were underrepresented in this respect. |
| Data collection | Online survey administered by Qualtrics<br>Workshops: in-person workshops in four locations. Data collected using audio recorders and paper questionnaires. The workshops included two researchers per breakout group (four researchers in total in the plenaries). All data anonymised prior to analysis. |
| Timing | June-December 2023 |
| Data exclusions | |

| | |
|---|---|
| Data exclusions | Survey: 1883 responses excluded due to failure of quality checks: removal of duplicates, bot detection, removal of those with incorrect location or IP data, and failed attention checks within the survey itself |
| Non-participation | Workshop: 12 participants dropped out before the start of data collection (no-shows) |
| Randomization | Workshop participants were split into breakout groups in advance of the workshop, using their demographic data they had provided, based on achieving as much demographic balance as possible (with relevant caveats re. the small sample size, discussed in the manuscript). |

# Reporting for specific materials, systems and methods

We require information from authors about some types of materials, experimental systems and methods used in many studies. Here, indicate whether each material, system or method listed is relevant to your study. If you are not sure if a list item applies to your research, read the appropriate section before selecting a response.

## Materials & experimental systems

| n/a | Involved in the study |
|---|---|
| ☒ | ☐ Antibodies |
| ☒ | ☐ Eukaryotic cell lines |
| ☒ | ☐ Palaeontology and archaeology |
| ☒ | ☐ Animals and other organisms |
| ☒ | ☐ Clinical data |
| ☒ | ☐ Dual use research of concern |
| ☒ | ☐ Plants |

## Methods

| n/a | Involved in the study |
|---|---|
| ☒ | ☐ ChIP-seq |
| ☒ | ☐ Flow cytometry |
| ☒ | ☐ MRI-based neuroimaging |

## Plants

| | |
|---|---|
| Seed stocks | n/a (not sure why this 'plants' section is here since we didn't use any plants; there's an error in this smart pdf) |
| Novel plant genotypes | n/a |
| Authentication | n/a |
